# Pulmonary Macrophage and Dendritic Cell Responses to *Cryptococcus neoformans*

**DOI:** 10.3389/fcimb.2020.00037

**Published:** 2020-02-11

**Authors:** Benjamin N. Nelson, Ashlee N. Hawkins, Karen L. Wozniak

**Affiliations:** Department of Microbiology and Molecular Genetics, Oklahoma State University, Stillwater, OK, United States

**Keywords:** *Cryptococcus*, pulmonary macrophages, pulmonary dendritic cell, primary phagocytes, innate phagocytes, macrophage subsets, dendritic cell subsets, phagocyte-*Cryptococcus* interaction

## Abstract

The fungal pathogen *Cryptococcus neoformans* can cause life-threatening infections in immune compromised individuals. This pathogen is typically acquired via inhalation, and enters the respiratory tract. Innate immune cells such as macrophages and dendritic cells (DCs) are the first host cells that encounter *C. neoformans*, and the interactions between *Cryptococcus* and innate immune cells play a critical role in the progression of disease. *Cryptococcus* possesses several virulence factors and evasion strategies to prevent its killing and destruction by pulmonary phagocytes, but these phagocytic cells can also contribute to anti-cryptococcal responses. This review will focus on the interactions between *Cryptococcus* and primary macrophages and dendritic cells (DCs), dealing specifically with the cryptococcal/pulmonary cell interface.

## Introduction

*Cryptococcus neoformans* is a ubiquitous environmental fungal organism that primarily inhabits soil and bird excrement, and this organism can cause opportunistic infections in humans (reviewed in Kwon-Chung et al., 2014). Initial interactions of immune cells with *Cryptococcus* usually occur in the lung following inhalation of cryptococcal spores or basidiospores (reviewed in Ellis and Pfeiffer, 1990; Perfect and Casadevall, 2002). In recent years, there have been many published studies examining the intracellular parasitism of macrophages by *Cryptococcus* (reviewed in Coelho et al., 2014; Mansour et al., 2014; Leopold Wager et al., 2016; Heung, 2017), and there have also been several published studies examining the anti-cryptococcal capabilities of dendritic cells (DCs) (reviewed in Wozniak, 2018). Many studies examining macrophage-*Cryptococcus* interactions have been performed with cell line macrophages, which has yielded valuable information on the cryptococcal factors involved in intracellular growth of *Cryptococcus* in macrophages. However, as we will discuss in this review, primary macrophages and DCs (non-immortalized cells derived from humans or mice), and particularly those from the pulmonary tissues (which originate from the fetal yolk sac and liver before the onset of hematopoiesis) (Guilliams et al., 2013; Hoeffel et al., 2015; Cybulsky et al., 2016), can have different interactions which lead to different outcomes with this organism. Few studies have examined these phenomena in primary phagocytic populations from mouse or human cells, and even fewer have examined these in primary phagocytic cells derived from the pulmonary tissues of mice or humans. Recent literature has identified multiple subsets of both macrophages and DCs present in the pulmonary compartment of both mice and humans (reviewed in Guilliams et al., 2013; Misharin et al., 2013; Gordon et al., 2014; Desch et al., 2016; Patel et al., 2017; Hoffmann et al., 2018), and studies are beginning to show that these subsets have functional differences in their interaction with pathogenic microorganisms (Patel et al., 2017; Huang et al., 2018), which is now influencing the way that we view interactions of these cells with pulmonary pathogens such as *Cryptococcus*. In this review, we will summarize the current literature examining primary mouse and human macrophage and DC interactions with *Cryptococcus* as well as discuss the future of identifying mechanisms of cryptococcal resistance or susceptibility in each of these phagocytic subpopulations.

## Murine Macrophage/*Cryptococcus* Interactions

### Murine Pulmonary Macrophages With *Cryptococcus*

Innate immune cells serve as the first line of defense against invading airway pathogens (Cheung et al., 2000; Margalit and Kavanagh, 2015; Espinosa and Rivera, 2016; Lloyd and Marsland, 2017; De Leon-Rodriguez et al., 2018). In the mouse lung, mononuclear cells, consting of monocytes, are recruited to the lung tissue and can differentiate into macrophages, and DCs during a cryptococcal infection (Wozniak et al., 2006, 2009; Yang et al., 2014; Heung and Hohl, 2019). In addition, it is now appreciated that monocytes can traffic to the pulmonary tissues and continue to be functional monocytes (without needing to differentiate into macrophages or DCs) (Jakubzick et al., 2013). Over the years, many studies have examined the role of murine pulmonary macrophages in response to *Cryptococcus* (Kawakami et al., 1994; Feldmesser et al., 2000; Kechichian et al., 2007; Osterholzer et al., 2009a; Hardison et al., 2010; Arora et al., 2011; Leopold Wager, 2014, 2015; Chen et al., 2016; Stepanova et al., 2018). As discussed later in this review, using mutant, or depletion mouse models along with mutant cryptococal strains can also yield different information about macrophage-cryptococcal interactions.

*Cryptococcus neoformans* can be a facultative intracellular pathogen *in vitro*, and *in vivo* and it can sometimes be found intracellularly inside of macrophages in infected tissues of mice (Hardison et al., 2010), or alternatively, murine macrophages can kill *C. neoformans* (Levitz et al., 1999; Feldmesser et al., 2000; Hardison et al., 2012; Leopold Wager, 2015; De Leon-Rodriguez et al., 2018). Therefore, macrophages are a major determinant of the outcome of a murine cryptoccocal infection (Shao et al., 2005; Kechichian et al., 2007; Alanio et al., 2011; Mansour et al., 2011; Sabiiti et al., 2014; Leopold Wager, 2015; Tenor et al., 2015; Johnston et al., 2016). Once inside macrophages, *C. neoformans* has the ability to impair mitochondrial function and alter protein synthesis (Ben-Abdallah et al., 2012; Coelho et al., 2015). *Cryptococcus neoformans* is also capable of a non-lytic form of exocytosis from macrophages, which begins with the formation of large vacuoles in the cytoplasm by which *C. neoformans* can exit (Ma et al., 2006; Alvarez and Casadevall, 2007). The ability of *C. neoformans* to surivive inside of phagocytic cells allows the pathogen to exhibit a trojan horse mechanism (Charlier et al., 2009) that enables the fungal pathogen to successfully cross the blood brain barrier through transendothelial pores contributing to cryptococcal brain invasion (Santiago-Tirado et al., 2017).

The ability of macrophages to contain a cryptococcal infection is dependent upon the specific type of macrophage activation (Arora et al., 2005; Muller et al., 2007; Osterholzer et al., 2009a, 2011; Voelz et al., 2009; Zhang et al., 2009; Hardison et al., 2010; Davis et al., 2013). Macrophages display phenotype plasticity which alters their functioning depending upon various environmental factors (reviewed in Mosser and Edwards, 2008; Shapouri-Moghaddam et al., 2018). During murine infections, macrophage-cryptococcal outcomes are dependent upon M1/M2 (classical/alternative) macrophage polarization pathways (reviewed in Leopold Wager and Wormley, 2014). Macrophage polarization status is constantly in flux, influenced by cytokines, and chemokines in the microenvironment (Davis et al., 2013; Ruytinx et al., 2018). Polarization states are influenced by various combinations of microenvironmental cues that allow for M1/M2 phenotype switching. These cues consist of the activation of signaling pathways due to the presence of specific stimuli, the activation of specific miRNAs, and the expression of certain genes (Wang et al., 2014). The classically activated macrophage phenotype (M1, induced by IFN-γ and other inflammatory chemokines) is associated with reduced fungal burden, enhanced fungicidal activity and the resolution of lung tissue inflammation (Davis et al., 2013; Ruytinx et al., 2018). This is opposed to the alternatively activated (M2, induced by IL-4 and IL-13) phenotype (Hardison et al., 2010; Davis et al., 2013; Leopold Wager, 2014, 2015), which is associated with being a reservoir for replicating cryptococci (Muller et al., 2007; Hardison et al., 2010). STAT1 dependent activation pathways are essential for M1 polarization as well as for *C. neoformans* fungicidial activity via the production of nitric oxide (Hardison et al., 2010, 2012; Leopold Wager, 2014, 2015). Infection of STAT1 KO mice and STAT1 conditional KO mice using an IFN-γ producing strain of *C. neoformans* resulted in increased fungal burden, increased M2 activation, and reduced anti-cryptococcal activity compared to WT mice (Hardison et al., 2012; Leopold Wager, 2014, 2015; Leopold Wager et al., 2018).

In mice, classically activated macrophages produce large amounts of nitric oxide, a main effector mechanism contributing to anti-cryptococcal activity (Zhang et al., 2009, 2010; Hardison et al., 2010, 2012; Leopold Wager, 2014, 2015). Pulmonary murine macrophages allow increased amounts of intracellular cryptococcal replication when isolated from mice deficient in iNOS, an enzyme necessary for nitric oxide production (Leopold Wager, 2015). In the murine lung environment, response to a *C. neoformans* infection results in a fluctuating Th1/Th2 cytokine response over time (Arora et al., 2011). A higher IL-4/IFN-γ ratio contributes to a greater polarization toward the M2 phenotype, whereas a higher IFN-γ/IL-4 ratio contributes to a greater polarization toward the M1 phenotype. Equal amounts of IL-4/IFN-γ lead to an M1/M2 intermediate macrophage phenotype (Arora et al., 2011). Intracellular cryptococcal replication in murine alveolar macrophages induces phagolysosome damage. However, once the cells are polarized to the M1 phenotype via stimulation with IFN-γ, the ability of the pathogen to induce phagolysosome damage is abolished and the macrophages can successfully kill *C. neoformans* (Davis et al., 2015). While these findings relate to macrophage activation status, the initial interactions between lung macrophages (prior to activation to either the M1 or M2 phenotype) with *Cryptococcus* are currently unknown. This may be due to the presence of multiple macrophage subsets present in the naïve lung (Vermaelen and Pauwels, 2004; Gautier et al., 2012; Misharin et al., 2013; Zaynagetdinov et al., 2013; Tan and Krasnow, 2016; Gibbings et al., 2017) and our lack of knowledge in understanding the role(s) of each subset with *C. neoformans*. In addition, once infection is established, these populations can then also be influenced by cytokines and chemokines present in the pulmonary tissue microenvironment (reviewed in Leopold Wager and Wormley, 2014; Leopold Wager et al., 2016). Understanding which naïve phagocytic subset(s) are able to kill *C. neoformans* and which subset(s) allow intracellular growth will be important in understanding their role in either prevention of cryptococcal dissemination or enhancement cryptococcal dissemination of to the central nervous system (CNS).

### Murine Pulmonary Macrophage Populations

The murine pulmonary cavity consists of a heterogeneous population of macrophages which were identified by flow cytometry and gene expression profiling (Vermaelen and Pauwels, 2004; Gautier et al., 2012; Misharin et al., 2013; Zaynagetdinov et al., 2013; Tan and Krasnow, 2016; Gibbings et al., 2017). The distinct populations were identified as: alveolar macrophages, interstitial macrophages, monocyte-like Ly6C^+^ macrophages and monocyte-like Ly6C^−^ macrophages, each expressing unique cell surface markers (Misharin et al., 2013; Zaynagetdinov et al., 2013). In addition, the inflammatory monocyte population has also been defined in the pulmonary tissues (Palframan et al., 2001; Yang et al., 2014; Hey et al., 2016; Heung and Hohl, 2019). Alveolar macrophages are defined as CD11c^+^, CD11b^−^, F4/80^+^, SiglecF^hi^, ^CD24−^, CD68^hi^, Ly6C^−^, Ly6G^−^, and pulmonary interstitial macrophages are F4/80^+^, CD11b^+^, CD11c^lo^, Ly6G^−^, and MHC II^+^, CD14^lo^ Pulmonary monocyte-like Ly6C^−^ macrophages are F4/80^+^, CD11b^+^, CD11c^−^, Ly6G^lo^, MHC II^−^, CD24^−^ and Ly6C^−^, while the pulmonary monocyte-like Ly6C^+^ macrophages have the same markers but are Ly6C^+^ (Guilliams et al., 2013; Misharin et al., 2013; Zaynagetdinov et al., 2013; Kopf et al., 2015; Gibbings et al., 2017). The inflammatory monocyte population is defined as Ly6C^hi^, CD11b^+^, CD115^+^, CCR2^hi^ (Yang et al., 2014; Hey et al., 2016; Menezes et al., 2016). All macrophage and monocyte markers are summarized in Table 1. Although each population has not been extensively studied with *C. neoformans*, murine alveolar macrophages and interstitial macrophages as well as inflammatory monocytes have been examined.

**Table 1:** Markers for murine pulmonary macrophage and dendritic cell subsets.

**Marker**	**Alveolar Mac**	**Interstitial Mac**	**Ly6c+ Mo-like Mac**	**Ly6c- Mo-like Mac**	**Inflam mono**	**CD103 DC**	**CD11b DC**
CD45	+	+	+	+	+	+	+
CD11b	−	+	+	+	hi	−	+
CD11c	+	lo	+/−	+/−	−	+	+
CD68	hi	lo	−	−	−	hi	hi
MHC II	+/−	+	−	−	−	+	+
Siglec F	hi	−	−	−	−	−	−
F4/80	+	+	+	+	+	−	−
CD14	−	lo				−	−
CD24	−	−	−	−	−	+	+
CD103	−	−	−	−	−	+	−
Ly6c	−	−	+	−	hi	−	−
Ly6G	−	−			−	−	−
CD115	−	−	−	−	+	−	−
CCR2	−	−	+	+	hi	−	−/+

Murine pulmonary macrophages/monocytes are divided into 5 subsets and pulmonary conventional DCs are divided into 2 subsets based on cell surface expression of multiple markers (Guilliams et al., 2013; Misharin et al., 2013; Zaynagetdinov et al., 2013; Yang et al., 2014; Kopf et al., 2015; Hey et al., 2016; Menezes et al., 2016; Gibbings et al., 2017).

### Murine Alveolar Macrophages

Murine alveolar macrophages are the first line of defense against inhaled pulmonary pathogens (Todd et al., 2016; Xu and Shinohara, 2017). Alveolar macrophages are tissue resident macrophages of the alveoli that are derived during early fetal development from either the fetal yolk sac or fetal liver (reviewed in Hoeffel et al., 2015; Kopf et al., 2015). During early developmental stages, fetal monocytes colonize embryonic lungs, and upregulate surface expression of Siglec F and CD11c, and develop into tissue resident alveolar macrophages (Guilliams et al., 2013). After inhalation and phagocytosis of *C. neoformans*, alveolar macrophages showed intracellular cryptococcal cells, and electron microscopy of these lung macrophages indicated cell damage (Feldmesser et al., 2000). In mice lacking NK cells and T cells infected with a glucosylceramide deficient cryptococcal strain, Δ*gcs1*, depletion of alveolar macrophages by clodronate liposomes administered weekly via intranasal injection starting 48 h prior to an intranasal cryptoccocal infection improved mouse survival and decreased the dissemination of *C. neoformans* to the CNS. Though this is a highly modified system and differs greatly from wild type mice and wild type *C. neoformans*, these data suggest that the alveolar macrophages were involved in the dissemination of *C. neoformans* (Kechichian et al., 2007). A more recent study examined murine alveolar macrophage polarization following infection with high- and low-uptake clinical *C. neoformans* isolates, and showed that the alveolar macrophages from mice infected with the high-uptake strains had increased expression of M2-associated genes (*Arg1, Fizz1, Il13, and Ccl17*), while those from mice infected with the low-uptake strains had increased expression of M1-associated genes (*Nos2, Ifng, Il6, Tnfa, Mcp1, Csf2, Ip10*) (Hansakon et al., 2019), suggesting that cryptococcal strains may influence macrophage polarization.

### Murine Interstitial Macrophages

Although few studies have examined murine interstitial macrophages, one study showed that interstital macrophages harbored intracellular *C. neoformans* following intratracheal administration. In addition, transfer of infected pulmonary interstitial macrophages, that were collected by agitating extracted lung tissue with sterile 5-mm diameter glass beads and morphologically characterized by Giemsa-stained smears and flow cytometry, into recipient mice via the tail vein led to hematogenous dissemination of *C. neoformans* to the brain (Santangelo et al., 2004).

### Murine Inflammatory Monocytes

Murine inflammatory monocytes are precursors to macrophages and DCs that are recruited to the site of infection, and these cells express the chemokine receptor CCR2 (reviewed in Murray, 2018). Inflammatory monocytes are sometimes considered the precursor to macrophages and dendritic cells (Palframan et al., 2001; Yang et al., 2014), but other studies suggest that distinct monocyte subsets give rise to inflammatory monocytes and DCs (Menezes et al., 2016). Regardless of the eventual fate of these cells, several studies have shown that inflammatory monocytes are important in cryptococcal clearance. Cryptococcal infection of CCR2^−/−^ mice leads to Th2-type responses, increased lung fungal burden, and decreased recruitment of macrophages and DCs compared to WT mice (Traynor et al., 2000; Osterholzer et al., 2008, 2009a, 2011). In addition, in mice infected with *C. neoformans*, Ly6C^hi^ CCR2^+^ monocytes are recruited and can differentiate into fungicidal macrophages and DCs (Osterholzer et al., 2008, 2011). Interestingly, a recent study showed inflammatory monocytes are rapidly recruited to the lung during cryptococcal infection, but depletion of these cells using the CCR2 diptheria toxin receptor (DTR) system leads to improved host survival, reduced pulmonary fungal burden, and reduced dissemination (Heung and Hohl, 2019). These cells also upregulate genes involved in M2 macrophage polarization, suggesting that in this model, the inflammatory monocytes differentiate into M2 macrophages, leading to a more severe outcome during cryptococcal infection (Heung and Hohl, 2019).

## Murine DC/*Cryptococcus* Interactions

Dendritic cells (DCs) play an important role in controlling *Cryptococcus* infection (reviewed in Wozniak, 2018). DCs are recruited to the murine lung during a cryptococcal infection (Wozniak et al., 2006), and in a protective model of cryptococcal infection, more DCs are recruited to the lungs in protected mice compared to non-protected mice, suggesting an anti-cryptococcal role for DCs (Wozniak et al., 2009). Following DC-cryptococcal interactions, DCs mediate the adaptive immune response by presentation of antigen to *Cryptococcus*-specific T-cells (Wozniak et al., 2006). Upon cryptococcal phagocytosis by murine bone marrow-derived DCs (BMDCs), the fungal pathogen is intracellularly trafficked to the endosomal and lysosomal compartments where it is then degraded and destroyed by oxidative and non-oxidative mechanisms (Kelly et al., 2005; Wozniak and Levitz, 2008). The depletion of pulmonary murine DCs via the administration of diphtheria toxin (DT) to CD11c-DTR transgenic (Tg) mice leads to increased morbidity and mortality as well increased B cell and neutrophil accumulation which causes severe lung inflammation (Osterholzer et al., 2009b).

### Murine Pulmonary DC Subsets

In the murine lung, DCs are a heterogeneous population consisting of distinct subsets (Shortman and Liu, 2002; Condon et al., 2011; Misharin et al., 2013; Zaynagetdinov et al., 2013). This heterogeneous population consists of the CD11b^+^ myeloid DCs, plasmacytoid DCs (pDCs) and CD103^+^ DCs (Condon et al., 2011) (Table 1). CD11b^+^ DCs are characterized by their high expression of CD11c, MHC class II, and absence of CD103. pDCs are characterized by expression of CD11c, B220, and PDCA-1. Finally, CD103^+^ DCs are characterized by their expression of CD103, CD11c, MHC class II, and their lack of CD11b (Sung et al., 2006).

### Murine Pulmonary CD103^+^ DCs

The CD103^+^ population localizes in the lung mucosa and along the lung vascular wall (Leepiyasakulchai et al., 2013), however, few studies have examined these subsets in greater detail. Studies that have examined this cell type showed that of the DCs that infiltrated to the lung during cryptococcal infection, the CD103^+^ population was a very small proportion of total DCs (Osterholzer et al., 2009a; Eastman et al., 2015).

### Murine Pulmonary pDCs

Pulmonary pDCs infiltrate into the lungs during cryptococcal infection, and pDCs isolated from murine bone marrow have anti-cryptococcal activity (Hole et al., 2016). The recognition and uptake of *C. neoformans* by pDCs is dependent on expression of Dectin-3 and the chemokine receptor CXCR3, and the fungicidal activity of pDCs is attributed to the production of reactive oxygen species (ROS) within the lysosome (Hole et al., 2016).

### Murine Pulmonary CD11b^+^ DCs

Studies of cryptococcal infection models have shown that CD11b^+^ CD11c^+^ DCs traffic to the murine lung during cryptococcal infection and can present antigen to cryptococcal-specific T cells (Wozniak et al., 2006). CD11b^+^ DCs are required for clearance of *C. neoformans* from the lung (Osterholzer et al., 2009a). In addition, in a vaccine model of cryptococcosis, CD11b^+^ CD11c^+^ DCs infiltrate to the lungs during a protective immune response (Wozniak et al., 2009), and these cells are also capable of generating trained immunity memory-like responses toward the fungal pathogen (Hole et al., 2019).

## Human Macrophage/*Cryptococcus* Interactions

Human primary macrophage interactions with *C. neoformans* can be variable—in some studies, human macrophages have anti-cryptococcal capabilities, while in other others, *C. neoformans* can survive/replicate within human macrophages (Weinberg et al., 1987; Cameron et al., 1990; Levitz and Farrell, 1990; Vecchiarelli et al., 1994; Levitz et al., 1997, 1999). However, clinical studies have given some insight into human macrophage-cryptococcal interactions. Cryptococci can be observed within pulmonary macrophages in samples from patients with cryptococcosis (Gal et al., 1986). However, even in the presence of IFN-γ and inflammatory mediators, non-HIV patients with cryptococcal meningitis had M2 polarized macrophages and a poor prognosis (Panackal et al., 2015). This contrasts with the M1 skewing of macrophages during HIV-related cryptococcal meningitis with immune reconstitution, suggesting that human macrophage/cryptococcal interactions are more complex than what has been observed previously (reviewed in Barber et al., 2012). Macrophages themselves have a dual role within the immune system which includes both inflammatory (fungicidal) and non-inflammatory (tissue repair) functions (reviewed in Heung, 2017). The regulation and switching between the two activation phenotypes is controlled by cytokines in the local milieu (Davis et al., 2013). As discussed with murine macrophages, M1 or classical activation is thought to be protective against *C. neoformans*, while the M2 phenotype or alternative activation allows for cryptococcal survival (reviewed in Leopold Wager et al., 2016).

### Human Pulmonary Macrophages

Human pulmonary macrophages, like murine macrophages, also have distinct subsets within the lung, identified by cell surface markers and transcriptional regulation (Patel et al., 2017). These macrophage subsets consist of alveolar macrophages (AF^hi^, CD11c^+^), BDCA1^−^ CD14^+^ macrophages, and BDCA1^−^ CD14^−^ macrophages (Patel et al., 2017) (Table 2). Early studies examined human alveolar macrophages (AMs) and their interactions with *C. neoformans*. Weinberg et al. showed that human AMs had either fungistatic or fungicidal activity in response to an *in vitro* fungal challenge depending on the presence of serum. In order to be fungistatic, these resident phagocytes did not require stimulation, serum, or opsonizing antibody. However, for fungicidal activity, the addition of serum and phagocytosis by AMs was required (Weinberg et al., 1987). In other studies, human AMs inhibited cryptococcal replication, which requires serum but does not require activation of macrophages by L-arginine or endotoxin (Cameron et al., 1990). Furthermore, this activity did not produce any detectable levels of nitric oxide or arginase activity nor was it enhanced by IFN-γ (Cameron et al., 1990; Vecchiarelli et al., 1994). In contrast, another study concluded that unstimulated AMs could not effectively kill *C. neoformans* due a lack of phagosome-lysosome fusion (Vecchiarelli et al., 1994). AMs did induce proliferation of autologous T cells by antigen presentation through HLA-DR. However, the authors did note that T cell proliferation may be caused by memory T cell populations, due to the ubiquitous nature of the organism and presumed previous interactions between donors and *C. neoformans* (Vecchiarelli et al., 1994).

**Table 2:** Markers for human macrophage and dendritic cell subsets.

	**Alveolar macrophages**	**BDCA1^**−**^ CD14^**+**^ macrophages**	**BDCA1^**−**^ CD14^**−**^ macrophages**	**Langerin^**+**^ DCs**	**BDCA1^**+**^ CD14^**+**^ DCs**	**BDCA1^**+**^ CD14^**−**^ DCs**
Autofluorescence	hi	lo	lo	lo	lo	lo
HLA-DR	+	+	+	+	+	+
Langerin	−	−	−	+	−	−
CD11c	+	+	+	−	+	+
CD14	−	+	−	−	+	−
BDCA1	−	−	−	−	+	+

*Human pulmonary macrophages are divided into 3 subsets and pulmonary DCs are divided into 3 subsets based on cell surface expression of multiple markers (Demedts et al., 2005; Patel et al., 2017).*

### Human PBMC-Derived Macrophages

Due to difficulty in obtaining human pulmonary macrophages, only a few studies have examined their interaction with *C. neoformans*. Instead, the more readily available peripheral blood mononuclear cells (PBMCs) can be differentiated into macrophages *in vitro*. While not tissue resident lung immune cells, PBMC-derived monocytes and macrophages provide an approximation of pulmonary macrophage responses to a cryptococcal challenge. Some of the first work with human PBMC-derived macrophages and *C. neoformans* was in 1973 by Diamond and Bennet who showed that macrophages cultured from PBMCs were able to phagocytose the fungus but were unable to kill it effectively (Diamond and Bennett, 1973). In fact, these “poorly encapsulated” intracellular cryptococcal cells grew more rapidly when compared to an extracellular control (Diamond and Bennett, 1973). Later studies showed that the effectiveness of PBMC-derived monocytes to inhibit the fungus depends upon culture surface, opsonins, cytokines, and the presence of the polysaccharide capsule (Levitz and Farrell, 1990). For opsonization, binding to the capsule either by specific antibodies or with complement components is required. In the absence of cryptococcal-specific antibodies, fungal opsonization, and phagocytosis is mediated primarily through complement (Levitz and Tabuni, 1991).

Upon phagocytosis, the endosome containing *C. neoformans* fuses with the lysosome, which acidifies the pH of the compartment to ~5.0 (Levitz et al., 1999). This is unlike many other intracellular organisms that attempt to avoid this fusion and subsequent drop in pH. *Chlamydia, Coxiella*, and *Mycobacterium* and are among several different bacteria that escape the phagosome-lysosome fusion by different means and replicate intracellularly outside of the acidic lysosome (reviewed in Mitchell et al., 2016). However, during a cryptococcal infection, the phagolysosome goes through the normal maturation process and acidification that does not inhibit fungal growth (Qin et al., 2011). In fact, alkalinization with either chloroquine, or ammonium chloride markedly inhibited the growth of both intracellular cryptococcal cells and free-living cryptococcal cells in media (Levitz et al., 1997). Interestingly, more recent studies in human PBMC-derived macrophages have shown that *C. neoformans* can prevent maturation and complete acidification of the phagosome, calcium flux, and protease activity, making the environment more favorable for cryptococcal growth (Smith et al., 2015). Although these studies seem contradictory, each had different criteria for describing acidification—the Levitz et al. paper described the phagosome as pH 5.0 (Levitz et al., 1999), the paper by Smith et al. posits that the phagosome is prevented from complete maturation/acidification (Smith et al., 2015), as the pH of the macrophage phagosome can go as low as pH 4.3 (Chen, 2002).

Escape mechanisms by *C. neoformans* from cell line macrophages, murine macrophages, and human macrophages were described in 2006 in which following non-lytic cryptococcal exocytosis (vomocytosis), both pathogen and host appear morphologically normal and continue to function (Alvarez and Casadevall, 2006; Ma et al., 2006). This function is mediated by the canonical Arp2/3 complex and is suppressed by the MAP kinase ERK5 (Johnston and May, 2010; Gilbert et al., 2017). Inhibition of ERK5 increases vomocytosis, while stimulation of ERK5 decreases vomocytosis (Gilbert et al., 2017). In addition, donor-to-donor macrophage variation has been observed in both intracellular proliferation rates as well as vomocytosis rates (Garelnabi et al., 2018). Interestingly, these variations did not depend on gender, cytokine profiles, or gene polymorphisms (Garelnabi et al., 2018).

## Human Dendritic Cell/*Cryptococcus* Interactions

While it has been known for some time that dendritic cells (DCs) are present throughout the human lung, they are difficult to study due the problems associated with acquiring them from healthy donors (Sertl et al., 1986; Demedts et al., 2005). As with human pulmonary macrophages, pulmonary DC subsets have been identified (Patel et al., 2017) (reviewed in Condon et al., 2011). Three subsets of conventional pulmonary DCs in a non-diseased lung have been identified by transcriptional profiling and flow cytometry as Langerin^+^ DCs, BDCA1^+^ CD14^+^ DCs, and BDCA1^+^ CD14^−^ DCs (Demedts et al., 2005; Patel et al., 2017) (Table 2). However, as with human macrophages, most human DC studies have focused on using PBMC-derived DCs with *C. neoformans*.

### Human PBMC-Derived DCs

Early studies focused on DC maturation and antigen presentation following phagocytosis of the fungus. Interaction of acapsular strains of *C. neoformans* with DCs induced surface expression of DC maturation markers including MHC class I and II and the costimulatory markers CD40 and CD83 (Vecchiarelli et al., 2003). Without the addition of an anti-GXM opsonizing antibody or complement, a fully encapsulated strain was unable to induce this DC maturation. This would prevent the subsequent T cell responses necessary for clearance of the pathogen. Engagement of additional receptors may contribute to the maturation of DCs and future investigations should examine this. Three cryptococcal mutants, lacking a visible capsule due to a defect in GXM synthesis (serotype A *cap10* and *cap59*, and serotype D CAP67), were also tested for DC maturation (Grijpstra et al., 2009). The *cap10* mutant was unable to induce DC maturation, while the *cap59* and CAP67 strains were able to induce DC maturation as shown by increased expression of the costimulatory markers CD80 and CD86 as well as increased production of IL-10 and TNF-α. Interestingly, the *cap59* mutant lost this ability when co-cultured with wild type *C. neoformans*, suggesting that intact GXM is not required to prevent DC maturation (Grijpstra et al., 2009).

Phagocytosis of the fungus with anti-GXM antibody occurs following recognition via FcγRII (CD32) and FcγRIII (CD16) (Vecchiarelli et al., 2003; Wozniak and Levitz, 2008). However, receptor binding alone is not enough to induce maturation. Phagocytosis via complement also induced similar maturation patterns; however, heat-treated serum abrogated this, indicating a role for the heat-labile complement components (Kelly et al., 2005). Additional cryptococcal surface molecules include mannosylated glycoproteins known as mannoproteins that make up a large portion of the fungal cell wall (reviewed in Mansour and Levitz, 2003; Levitz and Specht, 2006). Similar to the phagocytosis of acapsular *C. neoformans*, mannoproteins are also able to induce DC maturation by induction of CD80, CD86, CD40, CD83 and the inhibition of the CD14, CD16, and CD32 receptors (Pietrella et al., 2005). In addition, these DCs are induced to secrete IL-12 and TNF-α, which are both important in anti-cryptococcal responses (Pietrella et al., 2005). These mannoproteins are recognized by C-type lectin receptors such as DC-SIGN (CD209) and mannose receptor (MR) (CD206) (Mansour et al., 2006).

Similar DC maturation studies have been investigated with *Cryptococcus gattii*, which can cause fatal infections in otherwise healthy individuals. It has been shown that DCs can effectively phagocytose and kill *C. gattii* (Huston et al., 2013). However, DC maturation does not occur, preventing a T cell-mediated response and its anti-cryptococcal effects. This is marked by a lack of expression of MHC class II, CD86, CD83, CD80, and CCR7, and a lack of TNF-α production (Huston et al., 2013). Addition of recombinant TNF-α or other stimulation resulting in the production of TNF-α leads to the maturation of DCs and restoration of T cell responses. Similar to *C. neoformans*, an acapsular *C. gattii cap59* mutant was able to induce DC maturation and T cell responses (Huston et al., 2016). This maturation is dependent on signaling with TNF-α and p38 MAPK, but not ERK activation (as is the case with macrophages).

Upon recognition with either antibody or complement, phagocytosis by PBMC-derived DCs occurs through the conventional zipper mechanism (Wozniak and Levitz, 2008). Human PBMC-derived DCs can rapidly phagocytose *C. neoformans* and localize it to LAMP-1^+^ lysosomal compartments (Wozniak and Levitz, 2008). Providing evidence that contents of the human DC lysosome is fungicidal, purified DC lysosomal extract from human DCs can kill the fungus *in vitro* (Wozniak and Levitz, 2008). The exact molecules and mechanisms are not completely known, however, there is evidence that lysosomal enzymes such as human cathepsin B can kill *C. neoformans in vitro* (Hole et al., 2012). Antifungal activity of human PBMC-derived DCs is reduced by inhibitors of reactive oxygen species (ROS), and opsonized *C. neoformans* elicits the release of TNF-α but not IL-10 (Kelly et al., 2005). In addition, antifungal activity of blood-derived human plasmacytoid DCs (pDCs) is not dependent on ROS or on recognition by Dectin 3 (in contrast to murine pDCs), leaving the mechanism of anti-cryptococcal activity unknown (Hole et al., 2016).

### Future Questions and Conclusions

While much has been learned about phagocyte-cryptococcal interactions over the years using cell lines and non-pulmonary primary macrophages, and DCs, differences have been observed between cell lines, and primary cells, and even between primary cells (from bone marrow or PBMCs) and pulmonary macrophages, and DCs. Differences in the origin of cell types and even environmental difference in specific organs/tissues are far too great to ignore. Future studies will need to focus on subsets of pulmonary phagocytic cells in order to better understand the interactions between pulmonary phagocytes and the fungal pathogen *Cryptococcus neoformans*. Especially considering that different outcomes have been described with pulmonary phagocytic subsets of mice and human in regards to bacterial pathogens, it is important to identify the interactions of these subsets with fungal pathogens. Understanding these tissue-relevant primary macrophages and DCs and their interactions with *C. neoformans* may lead to the development of immunotherapies to combat this deadly fungal infection.

## Author Contributions

BN, AH, and KW contributed to the writing, editing, and revision of the manuscript.

### Conflict of Interest

The authors declare that the research was conducted in the absence of any commercial or financial relationships that could be construed as a potential conflict of interest.
